# Management of thoracoscopic thymectomy in a myasthenia gravis patient

**DOI:** 10.4103/0019-5049.76591

**Published:** 2011

**Authors:** Kavita Adate, Archana Shinde, Shalini Thombre, Kalpana Harnagle

**Affiliations:** Department of Anaesthesiology, Smt. Kashibai Navale Medical College and General Hospital, Narhe, Pune, India

Sir,

A 45-year-old male farmer was a diagnosed case of myasthenia gravis with evidence of an increased anti-Ach-R antibody titre of 5.64 nmol/L (N<0.25) and a significant detrimental response to the nerve conduction study. He was on tab. pyridostigmine 30 mg four times daily. The patient came to casualty with severe dyspnoea, dysphagia and grade III power in all four limbs. The patient was intubated and shifted to intensive care unit (ICU). He was on the PS-SIMV mode and received prednisolone and azathioprine in addition to pyridostigmine 60 mg QID. But weaning was difficult due to repeated respiratory muscle weakness, so the patient received intravenous (IV) IgG 0.4 g/kg for 5 days. Meanwhile, his computed tomography (CT) of the thorax and neck revealed an anterior mediastinal mass of size 2.8 × 1.6 × 4.9 cm, most likely thymoma. Due to poor response to medical management, the patient was electively posted for thoracoscopic thymectomy.

Routine preanaesthetic evaluation was done. The patient gave history of dyspnoea and dysphagia even for liquids; hence he was put on ryle’s tube (RT) feed and medications. He had ptosis and grade III power in all four limbs. His routine blood investigations, electrocardiogram (ECG), chest X-ray (CXR), 2D Echo, baseline arterial blood gas (ABG), and room Air oxygen saturation (SPO_2_) were normal. Pulmonary function test (PFT) could not be done. Breath holding time was <10 s. The patient was classified as Ossermann type III and was posted for surgery with informed risk of postoperative ICU stay and ventilatory support. On the day of surgery, the patient received his routine medications through Ryle’s tube. An IV line with 16-G cannula and right basilic central venous line were secured. The patient was premedicated with glycopyrrolate 0.2 mg, midazolam 1 mg, fentanyl 100 mcg, ranitidine 150 mg, metaclopramide10 mg and cefotaxime 1 g IV. The patient was induced with single-breath sevoflurane and propofol 100 mg IV. The larynx was sprayed with 10% xylocaine. The trachea was intubated with 39 F left sided portex double lumen tube (DLT). Anaesthesia was maintained with O_2_+ N_2_O + sevoflurane (1.2-2 MAC). Heart rate (HR), electrocardiogram (ECG), non invasive blood pressure (NIBP), oxygen saturation (SpO _2_) and end tidal carbon dioxide (EtCO_2_), neuromuscular response (accelerograph) and P-max were monitored. Surgery was performed in the left decubitus position with three ports of a video-assisted thoracoscope (VAT). Right lung isolation was provided during surgery. Surgery lasted for 75 min without significant blood loss. Postoperatively, intercostal drain (ICD) was inserted on the right side and 0.25% bupivacaine was infiltrated at the port site. The patient was shifted to ICU after changing DLT with an 8.5 cuffed single lumen portex tube.

Postoperatively, his requirement of pyridostigmine, azathioprine and prednisolone was increased almost double. After clinical assessment, the trial of extubation was done on the third postoperative day, which failed due to respiratory muscle weakness. The patient was reintubated and received IgG and assisted ventilation. Meanwhile, the histopathology report confirmed the presence of thymic tissue. The patient was tracheostomised (11^th^ day) and slowly weaned off over a period of 21 days. He was discharged on the 40^th^ postoperative day with 75% reduction in his medications and advised to follow up with Ach-receptor antibody titre after 3 months which was normal.

The main concerns for the anaesthesiologist during thymectomy are respiratory muscle weakness and preoperative anticholinesterase, as these interact with anaesthetic drugs, potentiate the vagal tone and produce copious bronchial secretions.[1]

Our patient had an acute onset, severe respiratory muscle weakness with a short period of remission after a high dose of pyridostigmine, immunosuppressants and IgG therapy. Recent reports stated that plasmapheresis may be used alone as a preparation to thymectomy.[2] It could have improved or prolonged the remission period in our patient. Our patient was not willing for plasmapheresis; hence thymectomy was planned.

We used propofol with sevoflurane for rapid induction and better jaw relaxation with an adequate depth for DLT insertion. Train of four (TOF) was maintained zero with the minimum alveolar concentration (MAC) 1.2-2 of sevoflurane. Low blood gas solubility coefficient, same uptake and elimination kinetics of sevoflurane in all age groups, good muscle relaxant property, nonalteration of A-V conduction time and precise and rapid adjustment of the anaesthetic depth make this agent suitable as the sole induction and maintenance agent in the case of short duration surgery where muscle relaxants are best be avoided. Kiran and others have reported that sevoflurane can be used as sole anaesthetic agent for thymectomy in myasthenia gravis[3] while the Ng study described that total intravenous anaesthesia (TIVA) with propofol and remifentanyl preclude the need of a muscle relaxant for VAT thymectomy.[4]

Positive predictors of prolonged postoperative ventilation are Ossermann type III and IV, previous history of respiratory failure due to myasthenia gravis and associated steroid therapy.[2,5] Our patient had all these predictors positive which itself explain his prolonged need of postoperative respiratory support and discharge with tapered doses of anticholinesterase after thymectomy.

We conclude that, balanced anaesthesia with propofol, fentanyl and sevoflurane for thoracoscopic thymectomy preclude the need for muscle relaxants. As far as stress and Ossermann type III are concerned, the possibility of prolonged postoperative recovery should be kept in mind and should be managed with patience.
